# High-Risk Cervical Human Papillomavirus Infections among Human Immunodeficiency Virus-Positive Women in the Bahamas

**DOI:** 10.1371/journal.pone.0085429

**Published:** 2014-01-23

**Authors:** Dionne N. Dames, Elizabeth Blackman, Raleigh Butler, Emanuela Taioli, Stacy Eckstein, Karthik Devarajan, Andrea Griffith-Bowe, Perry Gomez, Camille Ragin, on behalf of the African Caribbean Cancer Consortium

**Affiliations:** 1 Department of Medicine, Princess Margaret Hospital, Nassau, Bahamas; 2 Cancer Prevention and Control Program, Fox Chase Cancer Center – Temple University Health System, Philadelphia, PA, USA; 3 Department of Obstetrics & Gynecology, Princess Margaret Hospital, Nassau, Bahamas; 4 Department of Population Health, North Shore Long Island Jewish/Hofstra School of Medicine, Manhasset, NY, USA; 5 Department of Epidemiology, University of Pittsburgh, Graduate School of Public Health and the University of Pittsburgh Cancer Institute, Pittsburgh, PA, USA; 6 Department of Biostatistics, Fox Chase Cancer Center, Philadelphia, PA, USA; Alberta Provincial Laboratory for Public Health/University of Alberta, Canada

## Abstract

**Background:**

High-risk (HR) HPV genotypes other than 16 and 18 have been detected in a significant proportion of immunocompromised females. We aim to evaluate the frequency of HR HPV genotypes in a population of HIV-positive Caribbean women.

**Methods:**

One hundred sixty-seven consecutive, non-pregnant, HIV-positive females ≥18 years were recruited in this study. Each participant received a vaginal examination, PAP smear, and completed a questionnaire. DNA was extracted for HPV testing in 86 patients.

**Results:**

Mean age was 39.1 years for women positive for HR HPV and 43.1 years for women negative for HR HPV (*P* value  = 0.040). 78% (130/167) of the women had HR HPV infections; the prevalence of abnormal cervical cytology was 38% among women who were HR HPV-positive compared to women who were HR HPV-negative (22%). Fifty-one percent of the 86 women with available genotype carried infections with HPV 16 and/or HPV 18; genotypes of unknown risk were also frequently observed. Women who had a CD4+ count of ≤200 had 7 times increased odds of carrying HR HPV infection in comparison to women with CD4+>200.

**Conclusions:**

HR HPV infections in HIV infected females may consist of more than just HPV 16 and 18, but also HPV 52 and 58. Further studies are needed to determine whether HPV 52 and 58 play a significant role in the development of cervical cytological abnormalities in HIV+ women.

## Introduction

Human Immunodeficiency Virus (HIV) infection leads to a decline in both the number and function of CD4+ T cells. This deficiency in the immune system places the infected person at risk for other infections. For example, Human papillomavirus (HPV) is the most common sexually transmitted virus that infects the cervix and is the major etiologic agent for cervical cancer. Studies show that immunodeficiency increases HPV persistence thus increasing the risk of cervical cytology abnormalities and invasive cervical cancer [1], [2].

High-risk (HR) HPV types 16 and 18 account for the majority of all invasive cervical cancers worldwide (56.6% and 16%, respectively), while HPV 58, 33, 45, 31, 52, 35, 59, 39, 51 and 56 in order of decreasing prevalence account for the other 27.4% of all invasive cancers worldwide [3]. Similarly in the Caribbean, 56.8% of all invasive cervical cancers are attributed to HPV 16, and 6.7% to HPV 18; HPV 31, 45, 39, 51, 52, 56, 59 and 73 in order of decreasing prevalence account for the other 35.5% of cancers [4]. In 2006, a meta-analysis of HPV infections in HIV-positive women (n = 5,578) suggested that HPV 16 is less prevalent among HIV positive women with high-grade cervical lesions, and that other subtypes (11, 18, 33, 51, 52, 53, 58 and 61) are also substantially represented in this population [5]. At the time of the meta-analysis, however, no studies had been conducted in the Caribbean islands. In 2009, we conducted a study of cervical HPV infection in HIV-positive Caribbean women living in the Bahamas and observed that 46% of women with normal cervical cytology were infected with HR HPV, and that the prevalence was 100% for women with high-grade cervical lesions [6]. HPV genotyping was not evaluated in this study; therefore the type-specific prevalence and distribution of the various HPV types in HIV-positive women in the Caribbean population was still unknown.

HIV-positive persons treated with highly active anti-retroviral therapy (HAART) have reconstitution of the immune system following a successful lowering of HIV viral load and an increase in CD4+ T cell counts. These patients are no longer dying predominantly from opportunistic infections, but may be affected by other chronic medical illnesses and malignancies. A recent review by Adler *et al* [7] suggests that HAART has limited ability in decreasing HPV-associated cervical cancers in HIV-positive women. There are no studies addressing this issue conducted for HIV-positive women in the Caribbean.

In this study, the objectives are to evaluate the frequency and distribution of HR HPV genotypes in a population of HIV-positive Caribbean women and determine whether HR HPV infections other than HPV 16 and 18 are predominant in women with abnormal cervical cytology.

## Methods

### Study Population

The study received ethical approval from the Ethics Committee of the Public Hospital Authority and the University of the West Indies – Bahamas Campus. All participants provided written informed consent. Following Institutional Review Board approvals, 176 consecutive non-pregnant, HIV sero-positive females 18 years or older were recruited from the Infectious Disease Clinic at the Princess Margaret Hospital (PMH) in New Providence, Nassau, Bahamas from February to September, 2008. Cervical samples were collected through a PAP smear from all women at the time of the visit, and DNA was extracted for HPV testing. Nine women were excluded from the study due to insufficient quality DNA; therefore, the final study population consisted of 167 women. The first 80 patients were part of a previous publication [6]. All women had a positive HIV status via ELISA and Western blot methods; none of the women reported having used IV drugs in their life-time.

The Infectious Disease Clinic at PMH caters to all the Bahaman Islands where the majority of persons (70%) reside in New Providence, Nassau and each year about 1,500 HIV-positive women are seen. This clinic's mandate is to cater to and treat all patients regardless of immigration status, nationality or finances by providing access to antiretrovirals and care. As a result, a small percentage of patients from other Caribbean countries are also seen at the clinic. Our study population reflects this demographic, whereby 93% (n = 155) of women are from Bahamas, while 7% are from other Caribbean countries (such as Haiti (5%, n = 9) and Jamaica (2%, n = 3)).

### Data collection

Each participant received a vaginal examination, Pap Smear and completed a questionnaire administered by an interviewer. The questionnaire collected demographic information, tobacco and alcohol use, reproductive information such as number of pregnancies, age at first menses, number of sexual partners, age at first intercourse, history of sexually transmitted infections.

Clinical information was collected for each participant and included the number of years diagnosed with HIV, number of years on HAART and compliance with therapy. In the Infectious Diseases clinic, patients were initiated on HAART when their CD4 count was less than 350 cells/μL. For all women, results from HIV testing (ELISA and Western Blot), CD4 counts (Flow Cytometry), HIV viral load (Polymerase Chain Reaction (PCR)) and HR HPV status (Hybrid Capture II (HC II)) were recorded in the patient records in a clinical data base. The HIV testing, viral load and CD4 counts were performed in the clinical laboratory at the Princess Margaret Hospital. The HC II assay was performed in a clinical laboratory at LabCorp in the USA (Miami, Florida). Review of patient records was performed to obtain these results for inclusion in this study. The participant's last CD4 count and HIV viral load were recorded. These tests were performed either at the time the cervical sample was obtained or within the previous two months.

### HPV testing

The HC II assay is capable of identifying HR HPV infections (HPV: 16, 18, 31, 33, 35, 39, 45, 51, 52, 56, 58, 59, and 68), results are reported as “detected” or “not detected” and were available from the medical records for 81 patients in this study. For the remaining 86 patients, cervical thin-prep samples were shipped to the United States (Ragin laboratory) where HPV genotyping was performed using the HPV Linear Array protocol. To confirm that the extracted DNA samples were PCR amplifiable, the β-globin gene fragment was amplified using PCO4 and GH20 PCR primers. All DNA extraction and PCR set-up were performed in a PCR-clean area separate from the post-DNA amplification areas in the laboratory. The specific HPV types in each cervical specimen were identified using a Linear Array HPV genotyping protocol as previously described [8]. Briefly, the linear array assay included a PCR-based protocol involving biotin-labeled primers which detected the 37 most common HPV genotypes identified in cervical mucosa. The PCR products were denatured and hybridized to strips containing HPV-specific oligonucleotide probes. A colorimetric change indicated the presence of a given HPV type. The HPV types identified by the Linear Array method includes (Low-risk: 6, 11, 40, 42, 54, 61, 70, 72, 81, CP6108; High Risk: 16, 18, 31, 33, 35, 39, 45, 51, 52, 56, 58, 59, 68, 73, 82; Probable High Risk: 26, 53, 66; and Unknown Risk: 55, 62, 64, 67, 69, 71, 83, 84, IS39).

Both the Linear Array and HC II assays are comparable for HR HPV status since each detects the same HR HPV genotypes which are classified based on the previously published risk classification by Munoz *et al*. [9]. Therefore the results from both assays were combined for HR HPV analyses. The Linear Array additionally detects HPV 73 and 82 which are also classified as “high-risk”. Patient samples that were HCII-positive or had at least one HR HPV type detected by the Linear Array were categorized as HR HPV-positive. Patient samples with “probably high-risk” HPV genotypes were often co- infected with one or more other HR HPV genotypes (14/16, 88%) therefore were also categorized as HR HPV-positive. Since low-risk HPV types are not etiologically liked to cervical carcinogenesis [10] and there is no clinical benefit for evaluating low-risk HPV status for cervical cancer risk, study participants who were HPV-negative and/or infected with low-risk HPV types, and/or infected with undetermined risk were categorized as HR HPV-negative. Women with HR HPV findings were counseled and referred for usual clinical care; similarly, women with cervical lesions were referred to further testing and treatment.

### Statistical Analysis

Age was categorized into tertiles (19–35 years, 36–45 years, and 46+ years). Age at first intercourse was categorized as a binary variable based on mean age of responses to the question (≤16 years and >16 years). Current contraceptive use was categorized as yes/no. Length of time on HAART was categorized as less than 2 years, 2–4 years, and greater than 4 years. CD4+ cell count and viral load were both divided into two groups based on the median value for each respective variable. Cervical cytology reports were categorized into three categories: negative, dysplasia (which includes low grade squamous intraepithelial lesion [LGSIL] and high grade squamous intraepithelial lesion [HGSIL]), and atypical squamous cells of undetermined significance (ASCUS).

Descriptive statistics were generated for demographic, behavioral, and clinical variables according to the presence or absence of HR HPV infection, using Fisher's exact test to determine statistically significant differences between the proportions for each variable. Multivariable logistic regression models were used to assess the association of demographic, behavioral, and clinical variables with HR HPV infections. Pair-wise association of each variable with HR HPV status was performed. Covariates were included in the models based on statistically significant associations with HR HPV status (p<0.05). For the subset of participants where specific HPV genotypes were available, multivariable logistic regression models were used to assess the association between demographic, behavioral, and clinical variables and the odds of having HR HPV types 16 or 18 versus any other HR HPV type. Statistical analysis was performed using STATA version 10.1 software (Stata LP, College Station, TX). A *P* value of ≤0.05 was considered statistically significant.

## Results

### Descriptive Statistics of Overall Study Population

The study population (n = 167) consisted of women, ages 19 to 71 years old (mean age: 40 years). Table 1 summarizes the demographic and behavioral characteristics of the women according to HR HPV status. The mean age of HR HPV-positive women was younger than that of HR HPV-negative women (39.1 years vs. 43.1 years; *P* value  = 0.040). Overall 78% (130/167) of the women had HR HPV infections, and the prevalence of abnormal cervical cytology was 38% (49/130) among women who were HR HPV-positive compared to women who were HR HPV-negative (22% (8/37)). Cervical dysplasia rather than ASCUS was more prominent irrespective of HR HPV status. Seventy-seven percent (38/49) of HR HPV-positive women with abnormal cervical cytology had dysplasia compared to 62% (5/8) HR-HPV-negative women with abnormal cervical cytology. As expected, a significantly higher proportion of women who carried HR HPV infections had HIV viral load ≥400 and CD4 count ≤200 compared to women who were HR HPV-negative. HR HPV-positive women were more likely to have had a history of pregnancy compared to HR HPV-negative women (*P* = 0.048).

**Table 1:** Summary of demographic and behavioral characteristics of Bahamas HIV-positive women according to their HR HPV status.

Characteristic	HPV HR Positive (%)	HPV HR Negative (%)	*P* valueφ
	***N = 130***	***N = 37***	
**Age (years)**			0.065
** 19–35**	51 (39.2)	7 (18.9)	
** 36–45**	43 (33.1)	16 (43.2)	
** 46+**	36 (27.7)	14 (37.8)	
**Age at 1^st^ intercourse (years)**			0.853
** ≤16**	70 (53.9)	19 (51.3)	
** >16**	60 (46.1)	18 (48.7)	
Current contraceptive use^*^			0.563
** No**	73 (58.4)	18 (51.4)	
** Yes**	52 (41.6)	17 (48.6)	
**Number of Pregnancies**			0.048
** 0**	9 (6.9)	0	
** 1–3**	63 (48.5)	13 (35.1)	
** >3**	58 (44.6)	24 (64.9)	
**Presently on HAART^†^**			1.000
** Yes**	105 (81.4)	29 (80.6)	
** No**	24 (18.6)	7 (19.4)	
**Number of Years on HAART^†^**			0.063
** <2 years^††^**	33 (31.4)	6 (20.7)	
** 2–4 years**	43 (41.0)	8 (27.6)	
** >4 years**	29 (27.6)	15 (51.7)	
**Number of Years known HIV-positive**			0.579
** <3**	51 (39.2)	11 (29.7)	
** 3–9**	47 (36.2)	15 (40.5)	
** >9 years**	32 (24.6)	11 (29.7)	
**Last Viral Load mL^−1^**			0.001
** <400**	56 (43.1)	27 (73.0)	
** ≥400**	74 (56.9)	10 (27.0)	
**Last CD4 Count cell/μL**			<.0001
** ≤200**	55 (42.3)	3 (8.1)	
** >200**	75 (57.7)	34 (91.9)	
**Cervical Cytology**			0.079
** Normal**	81 (62.3)	29 (78.4)	
** Abnormal^¥^**	49 (37.7)	8 (21.6)	

φ Fisher's exact test p-values; ^*^Data missing for 7 women; Yes: birth control and male condoms, No: abstinence, sterilization and no method of birth control used; ^†^Data missing for 2 women; ^††^Includes women who answered “yes” to being on treatment but also answered “0” to number of years on treatment; ^¥^Includes ASCUS, Reactive and dysplasia.

**Figure 1:**
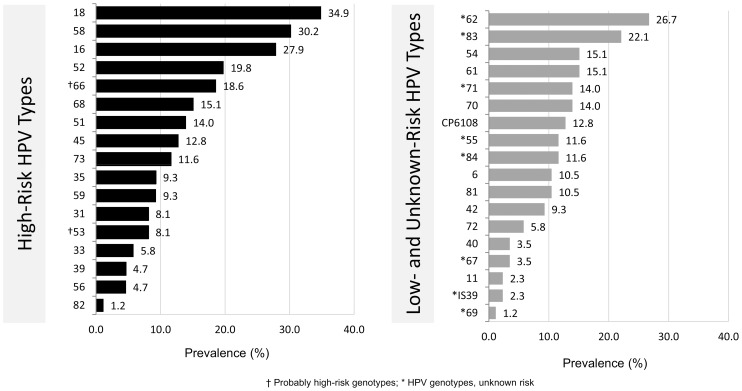
Genotype-specific distribution of high-risk and low/undetermined-risk HPV types detected in HIV-positive women. The prevalence does not total 100% since several women were infected with multiple HPV types.

Among the women with available HPV genotype data (n = 86), a wide distribution of HR and low/unknown-risk HPV types was detected (Figure 1). The prevalence of HR HPV types range from 34.9% to 1.2%, and HPV 18 was the most frequently detected. HPV 16 and 18 were the most prevalent in younger women, (≤39 years,) while HPV 18 and 58 were most prevalent in older women (>39 years) (data not shown). Similarly, the prevalence of Low-Risk HPV types (including genotypes of unknown risk) ranged from 26.7% to 1.25%; HPV 62, a genotype with unknown risk, was most frequently detected. Fifty-one percent of the 86 women with available genotype carried infections with HPV 16 and/or HPV 18. The majority, 73/86 (85%), carried multiple cervical HPV infections of any HPV type ranging from two to fifteen different HPVs in a single sample. With regard to HR HPV genotypes, women carried at least one or as many as 7 in any given sample.

### Multivariable Analysis

Women who had a CD4+ count of ≤200 had 7 times increased odds of carrying HR HPV infection (Adjusted Odds Ratio [Adj OR] 7.27, 95% Confidence Interval [CI] 1.41- 37.53-Table 2). There were no statistically significant associations between the other covariates and HR HPV infections (i.e. age, number of years on HAART, viral load and cervical cytology).

**Table 2:** Association between clinical factors and HR HPV infection (n = 167).

Variable	Adjusted Odds Ratio*	95% Confidence Interval
Number of Years on HAART		
<2 years	1.0	ref
2–4 years	1.54	(0.43–5.56)
>4 years	0.55	(0.17–1.82)
Last CD4 count cell/μL		
>200	1.0	ref
≤200	7.27	(1.41–37.53)
Last Viral Load mL^−1^		
<400	1.0	ref
≥400	1.62	(0.47–5.51)
Cervical Cytology		
Negative	1.0	ref
ASCUS & Reactive	0.74	(0.16–3.44)
Dysplasia (HGSIL or LGSIL)	1.75	(0.42–7.32)

* Adjusted for age, length of time on HAART, CD4+ count, viral load, and cervical abnormalities.

Among the 86 women for which specific HPV genotype data were available, 76 had HR HPV infections. For these women, after adjusting for possible confounders, women with a CD4 count ≤200 cell μ/L appeared more likely to be HPV 16/18 positive compared to women with a higher CD4 count, but this association was not statistically significant. No other variable was associated with carrying an HPV 16/18 infection in comparison to having another HR infection (Table 3).

**Table 3:** Association between clinical factors and infections with HR 16 and/or 18 vs. other HR genotypes (n = 76).

Variable	Odds Ratio*	95% Confidence Interval
Number of Years on HAART		
<2 years	1.0	ref
2–4 years	0.76	(0.14–4.07)
>4 years	0.53	(0.09–3.28)
Last CD4 count cell/μL		
>200	1.0	ref
≤200	2.98	(0.60–14.64)
Last Viral Load mL^−1^		
<400	1.0	ref
≥400	0.81	(0.15–4.27)
Cervical Abnormalities		
Negative	1.0	ref
ASCUS & Reactive	–	–
Dysplasia (HGSIL or LGSIL)	0.28	(0.03–2.41)

* Adjusted for age, length of time on HAART, CD4+ count, viral load, and cervical abnormalities.

## Discussion

In this sample of HIV-positive Caribbean women, we report that HR types 16 and 18 were among the most commonly diagnosed HPVs, and that infections with HR HPV types 52 and 58 are frequent. Other studies involving HIV-positive women in the US and Africa have reported a high prevalence of HR HPV types other than HPV 16 and 18, and our study among Caribbean women is in line with these results. Sherestha *et al* noted an increased incidence and prevalence of HPV 58, 53/66, 68/70 and 31/33/35, in African American HIV-positive females [11]; these subtypes were more frequent than HPV 16 and 18. Similarly, Luque *et al* [12] determined that high-risk types other than 16 and 18 were the most prevalent in a diverse population of HIV-positive women in Rochester, New York. Types 56 and 53 occurred most frequently in that population, followed by HPV 16, 58, and 52. A Ugandan study with an HIV-positive female population conducted by Banura *et al* [13] revealed high-risk types 52, 33, 51, and 16 in this order. Finally, in Zambia, 145 HIV-positive women were evaluated and HPV 52, 58 and 53 was the most prevalent while HPV 16, 35 and 45 were less common in this study population [14].

The majority of women in our sample carried multiple HR HPV infections; this could be attributed, among others, to the immunosuppressive state due to HIV infection. Patel *et al*. reported that the risk of abnormal cervical cytology was increased among women who carried multiple HPV infections (OR = 6.0; 95% CI: 2.3–15.7) [15]; this may be partly attributed to a high prevalence of HPV infection, and to an increased likelihood of persistent infection due to HIV status [16], [17], [18], [19]. Studies show that infections with multiple high-risk HPV types may act synergistically in cervical cancer development [20]. Therefore, the recommendation of routine cervical cancer screening in HIV-positive women is appropriate. At the time of this study cervical cancer screening, although available through the public clinics, was not routinely performed as part of the Infectious Diseases Clinic's Programme. Since the completion of this study, the Gynecology department and HIV clinics at the Princess Margaret Hospital have revised their policies and now encourages HIV-positive women to have their Pap Smears at least annually.

Another interesting finding is that 78% of the women carried HR HPV subtypes, but only 38% presented with abnormal cervical cytology; this suggests that the majority of these infections were likely transient rather than persistent, or that these were new infections. In addition, a high rate of cervical abnormalities was observed among women without evidence of HR HPV types yet cervical dysplasia rather than ASCUS was more frequently diagnosed. Assuming that HPV remains the main risk factor for cervical cancer in the Caribbean, this finding may indicate that other HPV types, considered low risk, are actually responsible, alone or in combination with specific environmental/behavioral factors, for the observed cervical abnormalities.

There is limited epidemiologic evidence of a specific role of HPV 52 and 58 in cancer development in HIV-positive women. Although high-risk HPV types 16 and 18 are known to be associated with more than 70% of all cervical cancer cases, few studies suggest that this may not necessarily hold true for immunocompromised populations of non-European descent. Sahasrabuddhe *et al*. screened over 145 HIV-positive Zambian women and detected squamous cell carcinoma in 28 females. Thirteen (46.4%) SCC cases were positive for HPV 52, and 10 (35.7%) cases were positive for both HPV 58 and 16 [14]. Similarly, Maranga *et al*. reported a lower prevalence of HPV 16 and 18 in HIV-positive women with invasive cervical carcinoma compared to HIV-negative women with invasive cervical carcinoma [21]. In the present study, none of the study participants had invasive cervical carcinoma; therefore, associations between HPV52 and 58 with SCC cannot be explored in this sample. However, the next step will be to follow prospectively these women to determine whether these HR HPV types are associated with cancer development. This has public health relevance since the current vaccines only target types 16 and 18, thus offering little protection if other HR HPV types are shown to play a role in the immunocompromised population.

Our study has several limitations: despite the fact that we enrolled all consecutive patients from the Clinic, we cannot exclude that some selection bias occurred, thus we cannot affirm that the results have external validity for the entire HIV positive community in the Bahamas. In addition, only a subset of the study population had available type-specific HPV information. Another limitation in this study is that, given the retrospective nature of the design, the participant's last CD4 count and HIV viral load were either performed at the time of enrollment or within the previous two months. In the first month after initiating HAART, CD4 count will start to rise, and will continue to rise for the first three years on average before reaching a plateau. It is possible that the levels of CD4 and viral load may be inaccurate in patients where CD4 and viral load data were obtained prior to enrollment, and HAART was initiated at the time of enrollment.

Another limitation of this study is that we examined exfoliated cervical cells rather than cervical biopsies. A recent study compared HPV prevalence and genotype infections between exfoliated cells and the corresponding cervical biopsy from women who HIV-positive [22]. Among women diagnosed with CIN2/3, the prevalence of HPV and multiple HPV types were similar in the biopsy compared to the exfoliated cells. This was in contrast to HIV-positive women with normal cervical cytology where the prevalence of HPV and multiple HPV types was higher in exfoliated cells compared to biopsy specimens. Another limitation is that the retrospective nature of the study does not allow us to assess a causal relationship between the length of time on HAART and the risk of developing abnormal cervical cytology.

Our findings support the need for follow-up of HIV-positive women in a longitudinal study in the Bahamas. The data generated from these efforts would provide important clinical information related to HPV persistence and the dynamics of multiple infections with HR HPV types in this population. Comparisons of HPV infections between cervical biopsies and exfoliated cells would provide meaningful information for the development of appropriate screening and HPV triage protocols needed for the evaluation and management of HPV infections in HIV-positive women.

## Conclusions

To our knowledge our work represents the first study of HR HPV infections in a unique population of Black HIV-positive women treated in the Bahamas. Our findings correlate with what has been reported in other populations. This information will need to be confirmed in larger studies but already warrants the significance of HPV screening in HIV-positive populations. Furthermore, the findings from our study could provide some insight on the potential efficacy of the current HPV vaccines in a population where HIV infection is wide-spread.
